# Current Technologies for RNA-Directed Liquid Diagnostics

**DOI:** 10.3390/cancers13205060

**Published:** 2021-10-10

**Authors:** Maria Victoria Martinez-Dominguez, Alja Zottel, Neja Šamec, Ivana Jovčevska, Can Dincer, Ulf Dietrich Kahlert, Ann-Christin Nickel

**Affiliations:** 1 Clinic for Neurosurgery, Medical Faculty, Heinrich-Heine University Düsseldorf, 40225 Düsseldorf, Germany; mariavictoria.martinezdominguez@med.uni-duesseldorf.de (M.V.M.-D.); ulf.kahlert@med.uni-duesseldorf.de (U.D.K.); 2 Medical Center for Molecular Biology, Institute of Biochemistry and Molecular Genetics, Faculty of Medicine, University of Ljubljana, 1000 Ljubljana, Slovenia; alja.zottel@mf.uni-lj.si (A.Z.); neja.samec@mf.uni-lj.si (N.Š.); ivana.jovcevska@mf.uni-lj.si (I.J.); 3 FIT Freiburg Center for Interactive Materials and Bioinspired Technologies, University of Freiburg, 79110 Freiburg, Germany; dincer@imtek.de; 4 Laboratory for Sensors, Department of Microsystems Engineering—IMTEK, University of Freiburg, 79110 Freiburg, Germany; 5 Molecular and Experimental Surgery, Clinic of General-, Visceral-, Vascular-, and Transplant Surgery, University Hospital Magdeburg, 39120 Magdeburg, Germany

**Keywords:** liquid biopsy, circulating free nucleic acids, exosome, cancer biomarker

## Abstract

**Simple Summary:**

The discovery of nucleic acids in blood has rapidly led into the development of methods for their isolation, characterisation and validation in the context of liquid biopsy. In this emerging field, miRNAs are promising biomarkers for the detection of solid tumours in the body fluids of cancer patients. This review gives an overview of the current methods of isolating circulating free and exosomal miRNAs, the advantages and disadvantages of the methods and the different techniques for miRNA quantification. At the end, the clinical relevance and market value of RNA-based liquid biopsy are explored and discussed with respect to the field of precision medicine.

**Abstract:**

There is unequivocal acceptance of the variety of enormous potential liquid nucleic acid-based diagnostics seems to offer. However, the existing controversies and the increased awareness of RNA-based techniques in society during the current global COVID-19 pandemic have made the readiness of liquid nucleic acid-based diagnostics for routine use a matter of concern. In this regard—and in the context of oncology—our review presented and discussed the status quo of RNA-based liquid diagnostics. We summarized the technical background of the available assays and benchmarked their applicability against each other. Herein, we compared the technology readiness level in the clinical context, economic aspects, implementation as part of routine point-of-care testing as well as performance power. Since the preventive care market is the most promising application sector, we also investigated whether the developments predominantly occur in the context of early disease detection or surveillance of therapy success. In addition, we provided a careful view on the current biotechnology investment activities in this sector to indicate the most attractive strategies for future economic success. Taken together, our review shall serve as a current reference, at the interplay of technology, clinical use and economic potential, to guide the interested readers in this rapid developing sector of precision medicine.

## 1. Introduction

Liquid biopsy is a simple and minimal invasive procedure alternative to surgical biopsies. It has gained relevance first as a method of analysing circulating tumour cells (CTCs) contained in blood and has since expanded to include the analysis of the materials secreted by the cells, which include circulating free nucleic acids (DNA, RNA) as well as exosomes [1]. The interest for liquid biopsy as a method for detecting cancer biomarkers has been increasing over the years as it presents several benefits to the gold standard method, the tissue biopsy. Tissue biopsy has been the main pillar of cancer diagnoses, but since it requires surgical operation and is sometimes limited by sample availability, newer non- or minimally invasive methodologies are currently being researched [2]. Amongst the main advantages of liquid biopsy is that it permits an easier sampling, which is sometimes the only option. It also allows a better profiling of the cancer genotype in the diagnostic and prognostic field because traditional tissue biopsies often fail to reflect the complete cancer gene expression profile. Besides, it fails to mirror the heterogenicity of the tumour that evolves over time [3]. Another advantage of liquid biopsy sampling is that it can be repeated over time, allowing for real-time tumour monitoring. Namely, early diagnosis of cancer relies on imaging techniques, mainly computed tomography scanning (CT) or magnetic resonance (MR). Although imaging remains the gold standard for solid tumour screening and monitoring, the emerging approaches have been reported to possess higher sensitivity, such as CTC and their released products, in combination with imaging techniques [4]. Similar is true for liquid tumour biopsies in which peripheral mononuclear blood cells (PBMCs) can be used for lymphoma, leukaemia and sarcoma diagnostics, amongst others [5].

Liquid biopsy therefore is a groundbreaking new alternative to the current decision making for targeted therapy in patients [6]. In addition, liquid biopsy can be used for cancer screening applications for early diagnosis or for prognosis of tumour metastasis leading to higher patient survival and tailored treatment [7].

## 2. Sources of Circulating RNA Biomarkers

**Figure 1:**
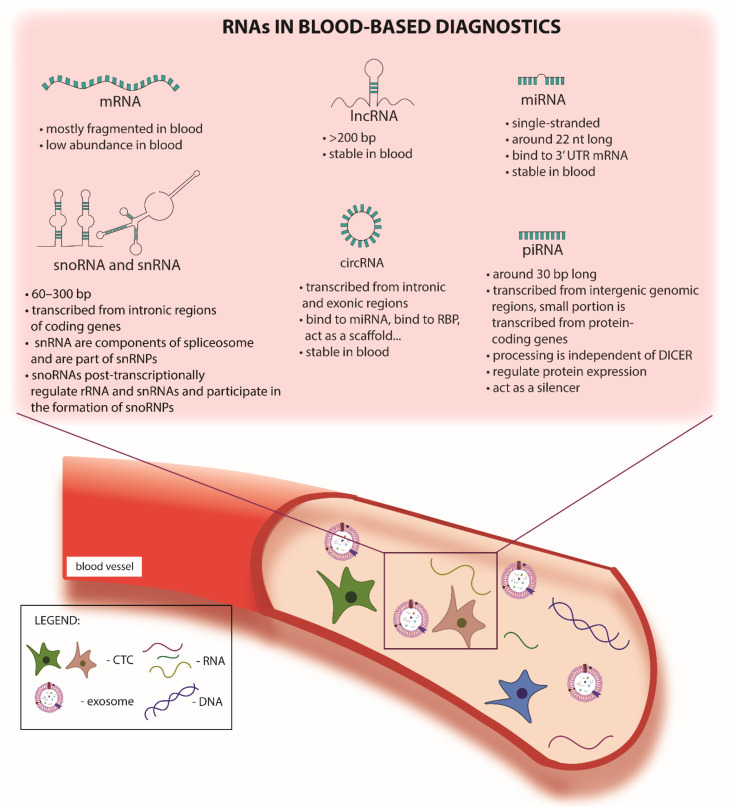
RNAs in blood-based diagnostics. The main types of RNAs found in blood-based diagnostics include mRNAs, long non-coding RNAs, microRNAs, small nucleolar RNAs, small nuclear RNAs, circular RNAs and piwi-interacting RNAs. Additional markers in blood cancer tumour cells, ct-DNAs, CTCs and exosomes amongst others can be found.

In liquid biopsy, the most commonly investigated sources of biomarkers are CTC, circulating free nucleic acids and exosomes (cell-free) (Figure 1). For clarification purposes, different sources of liquid biopsy biomarkers are shown in Table 1 which also explains the use of specific terms. We decided to use the term “circulating free” for both RNA and DNA found outside exosomes as well as “exosome” throughout the review.

### 2.1. CTC and cf-DNA

CTCs were first described in 1869 and are defined as cancer cells released by primary and/or secondary (metastatic) places that can be identified in the circulating blood [8,9]. They have been reported in different solid tumours giving information on the mutation profile of the tumour origin [10], therefore being among the first biomarkers to be detected for cancer purposes in liquid biopsy. However, the sensitivity of CTC detection, especially when searching for early stages of cancer, remains a challenge. Starting from a low number release, the majority of CTCs are eliminated via bloodstream (1 CTC/1 billion blood cells being present in the circulation at a certain time) and therefore analytical validity and detection techniques must be clearly established [11,12,13].

Circulating free DNA (cf-DNA) is formed of short fragments of around 160 base pairs. The portion that is originated from tumour cells is named circulating tumour DNA (ct-DNA) [14]. Because both cf-DNA and ct-DNA are stable in circulation, researchers followed the path of ct-DNA as a biomarker since it could provide a molecular profile of cancer [15]. Most studies focus on ct-DNA released in blood (plasma or serum) but ct-DNA molecules have also been found in different body fluids such as saliva, urine, pleural effusion or cerebrospinal fluid [16].

### 2.2. cf-RNA

Circulating free RNA are degraded and released RNA fragments to the bloodstream. Depending on the length, they can be divided into small RNAs (20–200 nucleotides) and long RNAs (>200 nt). Despite the fact that CTCs and ct-DNA have mainly led the research field of liquid biopsy, the focus has expanded to the analysis of cell-released RNA. Several cf-RNA types are highly abundant in the circulation, and since the majority of cf-DNA is released by necrotic or apoptotic cells, cf-RNA can give both information from apoptotic/necrotic tumour cells and from active tumour cells, such as exosome-mediated signalling, providing real-time information on the cancer [17]. However, the main inconvenience of cf-RNA is that it is not stable in the environment and could be degraded by ribonucleases, making their isolation problematic [18]. In spite of these limitations, the presence of extracellular cf-mRNA in the bloodstream of patients with cancer was confirmed in 1999 and led to the idea of mRNA as a cancer biomarker for prognostic and diagnostic value. Several articles have used mRNA expression panels to determine prognosis, metastasis and recurrence risk in different types of cancers [19,20].

Due to their higher stability and abundance, there is nowadays a great increase in the research done in non-coding RNAs (ncRNAs), particularly in small RNAs that could serve as diagnostic and prognostic biomarkers for disease [21]. However, before its successful integration to clinical practice, further analysis comparing rates between solid and liquid biopsy are needed [9]. The presence of different ncRNAs in various body fluids has led to the finding that ncRNAs may be more stable than mRNAs and that they could also serve as valid biomarkers [22,23,24]. Many studies have detected lncRNAs mainly in serum and plasma [25], but also in urine [26], saliva [27], bile [28] and gastric juice [29], and have been related to cancer profiles [30,31]. Particularly, microRNA (miRNA) is the most studied ncRNA due to its stability and dysregulation [32].

In the majority of human cancers, miRNA levels are altered, and its expression is tissue-specific. Consequently, they are promising biomarkers under study, and many cancer types can therefore be classified considering their miRNA profile [33]. In 2019, Drokow et al. summarized the main miRNAs with potential use as diagnostic biomarkers [34].

### 2.3. Exosomes

Extracellular vesicles are released from both eukaryotes and prokaryotes and are classified into three groups: exosomes, microvesicles and apoptotic bodies [35]. Each group is distinguished by its size and biogenesis [36]. Exosomes are the smallest of EVs, measuring 30–150 nm, and are formed as intraluminal vesicles inside multivesicular bodies (MVB) [35,37]. Exosomes were previously identified as the means to secrete cell waste [38]. Today, they are known as an important way of cell–cell communication [39]. Exosomes also contain a large spectrum of RNAs, which are mostly short and include miRNA, mRNA and other RNAs, which are protected by the lipid bilayer, also composed of different lipid molecules. It is hypothesized that under cellular stress conditions (which is also a hallmark of cancer), normal vesicular trafficking and the content inside the vesicles are altered, making them a suitable biomarkers of cancer cells.

Due to heterogenous cargo and reflection of parent cells, exosomes are rapidly emerging as a powerful source of biomarkers for cancer diagnosis, prognosis and therapeutic response [40]. Exosomes are abundant in the sample, can reflect the parent cell, are also present in almost all body fluids and are very stable, which makes them easier to store and transport [37,40]. Compared to free nucleic acids as biomarkers, the advantages of exosomal miRNA are especially protection from the environment and better representation of the original cell [41]. Exosomal miRNAs are attractive cancer biomarkers as they are protected from RNAse degradation, are stable for a longer period of time and resistant to freeze–thaw cycles [42].

## 3. Technologies in RNA Isolation and Detection

### 3.1. Blood Samples

#### 3.1.1. cf-RNA

When analysing cf-RNA from blood, centrifugation steps are necessary to obtain the plasma or serum fraction from which the miRNAs are isolated. The main steps are described in different studies. Since cf-RNA is a source of multiple RNA types as described above, some, such as cf-miRNA, are more stable than others. Cf-miRNA interacts with other biomolecules or packaging membrane-coated or membrane-free particles which protect them from the RNases present in the circulation [43].

#### 3.1.2. Exosomes

Exosomes as a source of biomarkers must first be reliably and successfully isolated from the blood sample. Several factors should be addressed, including the patient’s age, gender, ethnicity, body mass index, disease, medication, and food intake. The more detailed information regarding factors influencing the exosome release and content is described in [44]. Additionally, method of blood collection plays an important factor. Shear force may induce haemolysis and even gentle agitation can induce platelet-derived EV release [45]. When analysing blood exosomes, plasma is preferred over serum, because the platelet-derived EVs in serum may account for more than 50% of all isolated exosomes [44]. The anticoagulant should also be carefully chosen. Heparin is strongly discouraged from use as it may be associated with false-negative PCR results [46]. EDTA may also interfere with PCR, but to lesser extent than heparin [44]. EDTA reduces EV–blood aggregates and platelet-derived EV release compared to the citrate of heparin [45]. The acid–citrate dextrose coagulant has also been shown to be superior to EDTA, heparin and citrate in terms of prevention of in vitro vesiculation [47]. Today, several methods for exosome isolation exist, including ultracentrifugation, size-based methods, immunoaffinity-based methods and exosome precipitation [39].

### 3.2. Other Body Fluids

The majority of the most well-studied body fluids for both circulating free and exosome fractions are, beside blood, plasma and serum [48,49,50], saliva or urine [51,52,53,54]; other fluids, such as breast milk [55], cerebrospinal fluid [56], bronchial lavage, ovarian follicular fluid [57], tears [58] or semen [59], are under study as well.

The review from Bryzgunova et al., 2021 outlines the variety of methods of cf-miRNA isolation from different body fluids [43]. As in blood analysis, a centrifugation step is crucial to eliminate any cell debris that can interact with the RNA fraction. Even though blood samples are the most prevalent in exosome studies, research on other body fluids is increasing. Exosomes from different body fluids can be isolated using standard methods for exosome isolation [60,61,62]. In addition, it has to be taken into account that the amount of exosomes and their purity vary between body fluids. Because of the vast amount of body fluids and the increasing number of techniques for the isolation of cf-RNA, this review does not address specifically each body fluid.

### 3.3. Isolation of cf-RNA

RNAs in body fluids can be released from cells as free RNAs associated with proteins (mainly argonaute-2), but also with high-density lipoproteins (HDLs), or are present inside extracellular vesicles [63]. Analysis of liquid biopsies and the identification of noninvasive biomarkers in solid tumours has grown exponentially over the last few years. This has led to an increasing number of methods optimized for the purification of circulating free RNA from biological fluids, mainly plasma, serum and urine. Currently, there are many ready-to-use kits available for isolating ncRNAs taking into account the readiness of the process and the quality of the isolated RNAs (miRNAs, lncRNAs and circRNAs) as cf-RNAs.

Extraction methods of cf-RNA vary depending on the manufacturer and can be based on the use of the TRIzol reagent, silica membranes (e.g., Qiagen, NorgenBiotek) or magnetic beads (e.g., Life Technologies) [64,65]. Purity and quality of the isolated cf-miRNA largely affect the accuracy, reproducibility and reliability of its quantification. Methodological aspects regarding the efficacy, efficiency, automaticity and reproducibility of cf-RNA isolation aspects attract little attention, but the improvement of the methodology is what will lead to diagnostically validated procedures for cf-RNA isolation. The following methods describe isolation of cf-RNA which describe RNA that is not packed in CTCs and can be found encapsulated in exosomes or forming ribonucleoprotein complexes for protection. A summary of the methods for isolating cf-RNA described below is presented in Table 2.

#### 3.3.1. TRIzol

TRIzol™ is a ready-to-use reagent designed to isolate high-quality total RNA. Different types of commercial kits, such as Trizol LS (Invitrogen, Life Sciences, Carlsbad, CA, USA), nowadays use this phenol-based method for the isolation of cf-miRNA because of its main advantages: it is easy and fast to use and the whole procedure is low-cost. In addition, since one of its main disadvantages is the presence of ethanol-based contaminants, many protocols try to improve the isolation yield by combining the TRIzol reagent procedure with the use of columns for purification and miRNA enrichment [66].

#### 3.3.2. Silica Membranes

Silica matrices have revolutionized the procedures for RNA isolation both for research and commercial purposes. The affinity between negatively charged RNA and positively charged silica membranes (gel or glass) results in selective binding of the RNA while the rest of the components and chemicals of the sample are washed away [67]. On the other hand, magnetic beads use complementary hybridization by antigens, oligonucleotides, aptamers among others to pull apart RNA from the mixture. Both techniques show the advantage of a quick and efficient recovery of RNA, making them a good choice for automation, high-throughput applications and high sample processing rate.

In 2015, Brunet-Vega et al. performed a study acknowledging the problem of cf-RNA isolation, particularly in the case of miRNAs [68]. They analysed five commercially available kits for serum/plasma miRNA to determine whether the miRNA profiles vary between the isolation methods. The kits used were miRCURY RNA Isolation Kit Biofluids (Exiqon, Vedbaek, Denmark); Plasma/Serum Circulating and Exosomal RNA Purification Mini Kit (Norgen, Thorold, Ontario, Canada); NucleoSpin miRNA Plasma (MachereyeNagel, Düren, Germany); miRNeasy Serum/Plasma Kit (Qiagen, Toronto, Ontario, Canada); and Direct-zol RNA MiniPrep (Zymo Research, Irvine, CA, USA). It was concluded that all the kits were suitable for miRNA isolation from plasma/serum samples, and they had similar values in both RNA isolation and quantification Ct values in qRT-PCR. However, the study also states the great difficulties involved in miRNA measurement. From storage conditions to the fact that miRNAs in different body fluids can be found in concentrations as ng/µL and some fluorometry- and spectrometry-based techniques are unsuitable, therefore, novel state-of-the-art methods are needed [69].

In 2014, Hantzsch et al. performed a research study comparing different RNA isolation protocols regarding miRNA and mRNA quantity and quality recovered using PAXgene (Qiagen, Hilden, Germany) or Tempus (Life Technologies, ThermoFisher, Waltham, MA, USA) tubes (allowing instant preservation) [70]. RNA yields were highest using the Tempus Tubes and lowest using the PAXgene.

#### 3.3.3. Magnetic Beads

In a study conducted by Pezzi et al. in 2018, the potential impact of both DNA and RNA nucleic acid isolation based on the magnetic bead technology was evaluated. The two kits used were Dynabeads mRNA DIRECT (ThermoFisher, Waltham, MA, USA) for magnetic bead-based isolation and RNeasy Mini Kit (Qiagen, Hilden, Germany) for silica membrane isolation, detecting for both methods no significant loss compared to the control. Both techniques were effective in the isolation of RNA [71]. There are currently also patents stating the high recovery efficiency regarding the use of magnetic nanoparticles with cf-RNA and cf-DNA (Magnetic Nanoparticle, United States Patent Application 20200124592; kind code: A1) [72,73,74].

#### 3.3.4. Isolation of cf-miRNA

From small non-coding RNAs as a valuable source of RNA biomarkers, circulating free miRNAs are evaluated as the most promising biomarkers. However, the majority of the isolation protocols nowadays exist for total cf-RNA isolation, while several recent protocols from companies such as Qiagen or Norgen Biotek include the option to specifically isolate small RNAs as cf-miRNAs, providing that total RNA isolation is without loss of miRNAs or that there is selective purification of miRNAs in the pool [75]. Extraction of at least some cf-miRNAs is highly dependent on the extraction method which suggests incomplete dissociation of cf-miRNA from complexes and highlights a connection between the efficacy of isolation, miRNA sequence and type of biomolecules used for its isolation.

In the study conducted by Sourvinou et al. in 2013, they evaluated four different extraction protocols for circulating free miRNAs from blood [76]. After collecting the blood in EDTA tubes, it was centrifuged at 2000× *g* for 10 min at RT conditions. Afterwards, miRNA was isolated from the plasma. Synthetic miRNA from *Caenorhabditis elegans* was added to all the samples as an exogenous spiked-in control for sample-to-sample variation normalization. Once the plasma was obtained, it was subjected to a second centrifugation at 12,000× *g* at 4 °C for 15 min to remove all cellular debris. The four protocols for cf-miRNA extraction were as follows: standard extraction protocol using TRIzol LS (Invitrogen, Life Sciences, Carlsbad, CA, USA); miRNeasy mini kit (Qiagen, Hilden, Germany); mirVana PARIS kit (Ambion Inc., Life Sciences, Austin, TX, USA); and miRNA purification kit (Norgen Biotek, Thorold, ON, Canada). The researchers concluded that the TRIzol protocol gave the worst results in the percentage of miRNA recovery compared to the mirVANA PARIS kit. Among the commercially available kits evaluated in the study, the mirVANA PARIS kit featured the highest recovery yield and the most reproducible results.

In 2012, Li and Kowdley evaluated three different commercial miRNA isolation kits comparing performance and recovery yield miRNeasy kit (Qiagen, Valencia, CA, USA), the miRVana PARIS kit (Ambion Inc., Life Sciences, Austin, TX, USA) and the total RNA isolation kit (Norgen Biotek, Thorold, ON, Canada) following the manufacturer’s protocols and improvements. The miRNA fraction from total RNA was present in all the three kits [77]. The researchers concluded that Qiagen or Ambion kits had better miRNA recovery and yield than Norgen kits. As much as 67.1% and 66.7% of small RNA isolated with the Qiagen kit and the Ambion kit, respectively, were miRNA fractions, a ratio significantly higher than the one obtained with the Norgen kit (21.2%). In addition, the RNA in these studies was eluted in the same volume of solution in all the three kits; therefore, the concentration of miRNA would directly indicate the miRNA yield. Among the three kits we tested, the Qiagen kit provided miRNA with the highest concentration, 48.8 pg/μL, the Ambion kit—the second highest, 29.3 pg/μL, and the Norgen kit—the lowest, 11.7 pg/μL [22].

### 3.4. Exosome Isolation Methods

A brief summary of the methods for isolating exosomes described below is presented in Table 3.

#### 3.4.1. Ultracentrifugation

Ultracentrifugation is the most commonly employed method for exosome isolation and is used in 81% of all cases [78]. It consists of several steps of centrifugation in order to remove cells and cell debris which are followed by two rounds of ultracentrifugation at 100,000× *g* [79]. Ultracentrifugation is easy to use, affordable over time, moderately time-consuming, and large sample volumes can be used [39,45]. The main disadvantages, however, are co-isolation of impurities such as proteins, fusion of exosomes and their deformation [80,81]. Another important disadvantage of ultracentrifugation is substantial exosome loss [39].

A variation of ultracentrifugation is density gradient ultracentrifugation, which separates vesicles based on their density (exosome density being 1.08–1.22 g/mL) [45,79]. After ultracentrifugation, exosomes are collected from the specific layer(s) based on their density and are usually washed with another step of ultracentrifugation [45]. Isolation from density gradient is more time-consuming than standard ultracentrifugation, but the purity of exosomes is higher [45,82].

#### 3.4.2. Size-Exclusion Chromatography

With size-exclusion chromatography (SEC), vesicles are separated by their size, as pores capture smaller molecules and vesicles elute first [78]. It is performed relatively fast, but usually requires another step of ultracentrifugation to concentrate the sample [45]. It efficiently removes potential contaminants, such as plasma proteins and lipoproteins; however, a completely pure sample is difficult to achieve [45]. As reported by Baranyai et al. in 2015, albumin contamination is frequent as it is isolated in same fractions as exosomes [81]. Moreover, in 2020, Holcar et al. reported that exosomes isolated form sucrose cushion ultracentrifugation have considerably lower amounts of contaminants compared to size-exclusion chromatography [83].

#### 3.4.3. Immunoaffinity-Based Methods

Immunoaffinity-based methods exploit the presence of specific proteins on the surface of exosomes that can be captured with the corresponding antibody. Several commercial kits have been developed for this purpose, such as ExoMir (TM) Kit (Urbandale, IA, USA), ExoQuick^®^ Exosome RNA Column Purification Kit (System Biosciences, Palo Alto, CA, USA) or Total Exosome RNA & Protein Isolation Kit (ThermoFisher, Waltham, MA, USA) [39,84]. The sample is usually incubated with either antibody-coated latex or magnetic beads [45]. Compared to ultracentrifugation, exosomes isolated by immunoaffinity have considerably higher yields and specificity [39,85]. On the other hand, there are some reports showing that immunoaffinity separation yields lower amounts of exosomes compared to other methods because some markers are perhaps not represented or recognized on all vesicles [44]. The main disadvantages of immunoaffinity methods are the limitation to smaller sample volumes and, when applied as an additional purification step of some other isolation method, the whole process is very time-consuming. Furthermore, only a subpopulation of EVs is isolated, as there is currently no single universal marker known to identify exosomes [45]. Moreover, the kits can only separate exosomes from cell-free sample and the isolated exosome sample is usually eluted with a special buffer that may disturb further analysis [85].

In 2018, Stranska et al. compared the membrane affinity-based method (exoEasy; Qiagen, Hilden, Germany) and SEC (qEV columns; Izon science, Lyon, France) for exosome isolation from human plasma samples [86]. The results showed that the exosome samples isolated with SEC had higher purity compared to those isolated with exoEasy. However, the number of vesicles and the concentration of proteins were considerably higher when isolated by exoEasy, although at the expense of non-exosomal particles. The authors also analysed the lipid content in the samples. In general, the samples isolated with size-exclusion chromatography had a higher content of LDL, while the samples isolated with exoEasy had higher triglyceride-high lipoproteins. This has to be considered for the analysis of the vesicle content.

#### 3.4.4. Precipitation Methods

Exosome precipitation is based on applying water-excluding polymers, such as polyethylene glycol (PEG). PEG attracts water molecules and forces less soluble components out of the sample. Usually, the mixture is incubated overnight at 4 °C and the exosomes are isolated with centrifugation or ultrafiltration [39]. Currently, several commercial precipitation kits for exosomes exist [39]. Isolation with precipitation is simple, requires low sample volume and is cost-effective. Exosome precipitation is based on applying water-excluding polymers, such as polyethylene glycol (PEG). PEG attracts water molecules and forces less soluble components out of the sample.

In 2015, Lobb et al. compared ExoQuick (System Biosciences, Palo Alto, CA, USA; method based on precipitation), ExoSpin (Cell Guidance Systems, Babraham, UK); the method is based on the combination of precipitation and SEC) and SEC for plasma exosome isolation. The yield of exosomes was the lowest using SEC, but the purity of samples was the highest. Furthermore, the amount of albumin contaminants was considerably lower compared to ExoQuick and ExoSpin [87].

In 2017, Helwa et al. compared ultracentrifugation and three different precipitation commercial kits for exosome isolation from serum: miRCURY RNA Isolation Kit (Exiqon, Woburn, MA, USA), ExoQuick (System Biosciences, Palo Alto, CA, USA) and Total Exosome Isolation Reagent (TEIR; Life Technologies, ThermoFisher, Waltham, MA, USA). The number of exosomes isolated by differential centrifugation was considerably lower (up to 130-fold, depending on the starting volume) compared to commercial kits. All the commercial kits had a similar yield; only TEIR produced slightly higher yield compared to miRCURY. When RNA was isolated from exosomes, interestingly, there were no differences in the amounts of RNA between commercial kits and differential centrifugation [88].

In 2019, Macías et al. compared six commercial kits for serum exosome isolation: ExoEasy (Qiagen, Hilden; Germany) (membrane affinity-based method), ExoQuick (precipitation-based method from System Biosciences, Palo Alto, CA, USA), ExoSpin (column-based method from Cell Guidance Systems Babraham, UK), ME (peptide binding method), ExoQuick Plus (Cell Guidance Systems Babraham, UK) (polymer precipitation followed by immunoaffinity capturing—negative selection) and Exo-Flow (System Biosciences, Palo Alto, CA, USA) (polymer precipitation followed by immunoaffinity capturing the anti-CD63 antibody). ExoQuick, ExoQuick Plus and ExoSpin had the highest yield while the yield of ME was around 400 times smaller compared to those three methods. In exosome samples isolated by Exo-Flow and ME, no apolipoprotein B was detected, and among the exosomes isolated with ExoQuick Plus, only one sample out of five was positive in apoB. On the other hand, exosomes isolated by ExoQuick only had large amount of contaminants [84].

#### 3.4.5. Isolation of miRNA from Exosomes

Exosomal miRNAs are attractive cancer biomarkers as they are protected from RNAse degradation, are stable for a longer period of time and resistant to freeze–thaw cycles [42]. The disadvantage in analysing exosomal miRNA is especially the presence of exosomes secreted by other cells. This could be, however, evaded by isolating exosomes based on tumour biomarkers and detecting multiple exosomal miRNAs [42]. Prior to exosome analysis, haemolysis should be assessed since it may be related to elevated concentrations of certain miRNAs, such as miR-486-5p, miR-451, miR-92a and miR-16 [89]. Another point to consider is the potential contamination with non-exosomal RNA, for example, with RNA bound to the surface of exosomes, since it may cause false interpretation [36]. Moreover, miRNA is also carried by lipoproteins which particularly complicate the measurement as it is nearly impossible to obtain lipoprotein-free exosome samples with the existing methods [90,91,92]. The other possible sources of RNA contaminants are ribonucleoprotein complexes and viruses. In some cases, contaminant RNA may be degraded by treatment with RNAse together with proteinase. Another difficulty in exosomal miRNA analysis is very low amount of miRNA in exosomes. Namely, in 2014, Chevillet et al. studied the abundance of miRNAs in plasma exosomes. The results show that their amount is relatively low, only one copy of specific, otherwise abundant, miRNA per approximately 47,000 exosomes [93].

MicroRNA may be isolated from exosomes using different commercially available kits. Interestingly, using TRIzol, the most popular method for RNA isolation, might not be suitable for exosome isolation, as certain miRNAs, especially poor in the GC content, may be lost during the procedure [94]. In 2014, Cheng et al. isolated miRNA using the miRNeasy mini Kit (Qiagen, Hilden, Germany) from plasma exosomes isolated by ultracentrifugation. In the parallel plasma sample, they isolated exosomal miRNA directly from plasma using a Exosomal RNA kit (Norgen Biotek, Thorold, OT, Canada). Both methods were approximately equally-efficient and yielded 4.7 ng (1.2 mL plasma; ultracentrifugation; Qiagen) and 4.9 ng (1 mL plasma; Norgen Biotek) miRNA, respectively. However, using a Norgen Biotek kit, more contaminating non-exosomal RNAs were isolated compared to ultracentrifugation and miRNeasy [95].

Prendergast analysed the exosomal RNA amount isolated from archival sera using different commercial kits and adapted protocols [96]. Exosomes were isolated with either ExoQuick (System Biosciences, Palo Alto, CA, USA) or ultracentrifugation on 15% sucrose cushion followed by another step of ultracentrifugation. RNA was isolated using four different protocols: (1) TRIzol and RNeasy (Qiagen, Hilden, Germany), (2) RNeasy Mini kit (Qiagen, Hilden, Germany), (3) RNA precipitation and (4) AllPrep DNA/RNA Mini kit (Qiagen, Hilden, Germany). RNAs were successfully isolated by all methods, except for AllPrep DNA/RNA Mini kit. When exosomes were isolated by ultracentrifugation, there were no differences in the RNA amount between each protocol. On the other hand, the concentration of RNA from exosomes isolated with ExoQuick was the highest when using RNA precipitation, probably because all the other methods isolated only larger RNAs.

In 2017, Tang et al. analysed RNA isolated from serum exosomes [97]. Exosomes were isolated using ultracentrifugation, ExoQuick (System Biosciences, Palo Alto, CA, USA) and Total Exosome Isolation kit (ThermoFisher Scientific, Waltham, MA, USA). RNAs were isolated using either TRIzol-LS (exosome isolated by ultracentrifugation), SeraMir (Sytem Biosciences, Palo Alto, CA, USA); exosomes isolated by ExoQuick (System Biosciences, Palo Alto, CA, USA), HiPure Liquid RNA/miRNA kit (La Roche, Basel, Switzerland); exosomes isolated by ExoQuick, miRNeasy (Qiagen, Hilden, Germany); exosomes isolated by ExoQuick, using exoRNeasy (Qiagen, Hilden, Germany) directly from serum and by means of Total Exosome RNA Isolation (ThermoFisher Scientific, Waltham, MA, USA; used with TEI). The concentration of exosomes isolated using ultracentrifugation was the lowest, but the purity was higher compared to other methods. The amount of RNA was the highest when isolated with a HiPure Liquid RNA/miRNA kit, ExoRNeasy and TER. ExoRNeasy was superior in the yield of small RNAs compared to all the other methods.

In 2018, Buschmann et al. isolated exosomal RNA from patients’ serums using different methods for exosome and RNA isolation: (1) exoRNeasy (Qiagen, Hilden, Germany), (2) SEC and miRCURY (Qiagen, Hilden, Germany), (3) Exo-spin (Cell Guidance Sytems, Babraham, UK) and miRCURY RNA isolation kit (Exiqon, Woburn, MA, USA), (4) miRCURY Exosome Isolation kit (Exiqon, Woburn, MA, USA) and miRCURY Exosome RNA isolation kit (Exiqon, Woburn, MA, USA), (5) Ultracentrifugation and exoRNeasy (Qiagen, Hilden, Germany) [98]. The results indicated that the miRNA enrichment was highest using the miRCURY Exosome Isolation Kit and miRCURY RNA Isolation kit for biofluids and lowest using SEC. Furthermore, the number of isolated exosomes was highest using the miRCURY Exosome Isolation Kit. However, the number of contaminants was the highest in exosome samples isolated using miRCURY and ultracentrifugation and the lowest in exosomes isolated by size-exclusion chromatography.

### 3.5. RNA Detection and Quantification

A brief summary of all the detection and quantification methods described below is presented in Table 4.

#### 3.5.1. Direct Detection and Quantification of RNA

Quantification of miRNA after the initial isolation is a crucial step for all further downstream applications. The indicated assays demonstrating a high flexibility and adaptability to all downstream applications such as the ddPCR, NGS or qPCR or can be used as stand-alone detection method.

Common methods to quantify RNA directly for further downstream applications are fluorescence-based detection using a Qubit Fluorometer (Technologies) or spectrophotometer-based technologies Bioanalyzer (Agilent Technologies, Santa Clara, CA, USA), Tecan Infinite^®^ 200 PRO Nanoquant Spectrophotometer (Tecan, Maennedorf, Switzerland) or Nanodrop Spectrophotometers (Thermo Scientific, Waltham, MA, USA). One major disadvantage of the spectrometer-based methods is that they cannot discriminate between the different types of RNAs, therefore revealing false yield of miRNA. Specifically, below the concentration of 1 ng/µL Nanoquant and the Nanodrop Spectrophotometers revealed high variability in single measurements while other platforms remained in the reproducible range [120]. Similar variability could be detected by means of the bioanalyzer platform using different older chip-based assays. Newer small RNA analysis kits claim to better perform quantification of miRNAs between 6 and 150 base pairs [41].

Unlike these techniques, Qubit (Thermo Scientific, Waltham, MA, USA) employs specific fluorescent dyes selective for small RNA or miRNA over other forms of RNA, and therefore the concentration values obtained with this platform should correspond mainly to the portion of small RNA [41]. A broad range of RNA kits for highly selective RNA binding are offered, such as the Qubit RNA HS Assay Kit (from 250 pg/µL to 100 ng/µL), the Qubit RNA BR Assay Kit (from 1 ng/µL to 1 µg/µL) and the Qubit microRNA Assay Kit [121]. In conclusion, the Qubit assays appeared to be the most appropriate method to estimate miRNA content in human plasma samples, enabling very sensitive miRNA detection.

#### 3.5.2. RNA Sequencing

Next-generation sequencing (NGS) describes a number of different modern sequencing technologies. All of these technologies allow the sequencing of short or long reads of DNA or RNA in a high-throughput manner. The advantages of these systems are that they are much faster, display a high accuracy and are usually cheaper when analysing a large amount of samples than the previously used methods.

Over the past few decades, RNA sequencing has significantly progressed and mostly employs NGS technology. Using this technology, it is now possible to detect even miRNAs in individual cells by using single-cell RNA-seq [122,123,124]; miRNA-seq either uses enriched small RNAs, which are known for specific biases, or total RNA [125]. At the next step, a cDNA library is prepared. Due to the small miRNA size, usually, ligation or polyadenylation is performed to produce primer-binding sites for reverse transcription. By adding sequencing adapters, the cDNA library preparation is finished and ready for the sequencing step [124]. The average number of the times each nucleotide is read in the sequencing process refers to the depth in high-throughput method and is linked to the relative expression level. To detect novel miRNA, at least five million reads per sample are needed to provide enough statistical power. With ultra-deep sequencing, even mutations in miRNA which occur in small fractions of the sample could be identified. Firstly, in the process, short reads are analysed and interpreted to remove low-quality sequences and adapters. Then, with one of the software processes, reads are mapped onto the genome reference sequence. Platform-independent biases, computational infrastructure required for data analysis and data interpretation are the main disadvantages of this method [104].

#### 3.5.3. qPCR

Quantitative PCR (qPCR or real-time PCR) is based on the PCR cycle analysis allowing for quantification of the desired sample; qPCR-based analyses combine “traditional” endpoint detection PCR with fluorescent detection technologies based on different fluorescent dyes, such as SYBR Green or TaqMan of FRET probes, to gain specificity. The basic mechanism consists in the multiple amplification cycles in which cDNA (reverse-transcribed from the RNA sample) follows cycles of denaturation and renaturation followed by the extension of the complementary strand by a thermostable DNA polymerase, resulting in an exponential increase of the sample.

The length of miRNA is usually around 21–23 base pairs, so it is challenging to design conventional PCR primers which are typically 20 base pairs long. In general, two different groups of reverse transcriptions are known, universal and specific reverse transcription. During the reverse transcription step, the length of miRNA is extended by adding the poly A tail to the 3’ end of miRNA with poly A polymerase for universal reverse transcription or using miRNA-specific stem loop primer for specific reverse transcription [111,126]. The universal methods also include, beside the poly A tail, polyuridylation, ligation of the universal linker and combination of linker ligation and end tailing [111]. The advantage of this method is that the reaction happens in the same tube. However, it can cause high background noise.

In the specific reverse transcription method, linear primers, pincer probes and stem loop primers are used. The development of stem loop RT-qPCR techniques provides a novel method for accurate and sensitive detection of miRNA [111]. Stem loop primers bind to the 3’ end of mature miRNAs. The hybridization of the RT primer to the other RNAs and to pre-miRNAs is prevented because of the double-stranded stem structure trait. MicroRNAs are then reverse-transcribed to complementary DNA. The cDNA is than amplified and quantified using a TaqMan probe, miRNA-specific forward and reverse primer [104].

#### 3.5.4. ddPCR

Progress has been made over the last decade in the development of more sensitive PCR-based methods. Digital PCR and digital droplet PCR methods are highly sensitive and low-cost methods and the workflow is similar to TaqMan probe real-time PCR. They are able to target the amounts in samples that are below the limit of conventional qPCR methods [110]. In ddPCR, amplification occurs in each individual droplet (out of 20,000) which are then analysed to determine the fraction of positive ones. Analysis of positive droplets is estimated under the assumption of Poisson’s distribution. High sensitivity of low-abundance targets, high inhibitor tolerance in the sample reaction and quantification with no need in a reference gene are the main advantages of ddPCR [104].

There is an increasing research effort on the ddPCR technology for assessing and quantifying the presence of various miRNA from different body fluids to get accurate absolute quantification of cf-miRNAs [127,128].

#### 3.5.5. Microarray

Microarray technology is a powerful high-throughput method where known high-density immobilized DNA probes are hybridized to detect target miRNA [105]. Anyway, miRNAs properties which are its low abundance, short length (aggravated optimization of hybridization efficiency) and large difference in the GC content (different hybridization properties) are challenging for the application of microarray in expression analysis. To overcome problems in hybridization, more efficient nucleotide analogues have been designed. For example, locked nucleic acid (LNA) allows sensitive profiling of miRNA because of the capture probe’s enhanced melting [104] temperature. Agilent probes have a stem loop sequence attached to the miRNA complementary region. When miRNA hybridizes, it can base-stack with the duplex region of the stem. This increases the base pairing energy, allowing more stringent hybridization conditions. Exiqon probes, in contrast, are designed with LNA bases interspersed within the DNA sequence. This affects the helical structure of the hybrid and increases the base pairing energy [129] 

In a study conducted by Callari et al. in 2013, it was researched if microarrays can be accurately used for circulating miRNAs from plasma [130]. The reliability of miRNA quantification depends mostly on its strategy and, afterwards, its data normalization. 

## 4. Clinical Relevance and Economic Aspect of Circulating Biomarkers

### 4.1. Clinical Relevance of Emerging Liquid Biopsy

Liquid biopsy of circulating biomarkers has the potential to be developed as a minimally invasive diagnostic methodology for precision medicine, and its potential values have received increasing attention over the last decades [131,132,133]. Several traditional blood tumour marker proteins, such as CA15–3, CA19–9 and PSA markers are already regularly used in the patient management of respectively breast, colorectal, and prostate cancer [134]. In addition, PBMCs and their nucleic acid content have also been intensively researched as tumour biomarkers. In the studies conducted by Chang et al. and by Mishra et al. in 2015, dysregulation of several miRNAs was used as diagnostic and prognostic markers in breast cancer patients [135,136,137]. With the establishment of advanced detection technologies (such as NGS), cancer biomarkers at low concentrations can nowadays be studied in ct-DNA, cf-RNA, td-EV, miRNA and CTCs [41,134]. The massive potential of that method in clinics was shown by the US Food and Drug Administration (FDA) approving a first liquid biopsy companion diagnostic test, the Guardian360 CDx in 2020. This NGS-based technology identifies mutational variants of the epidermal growth factor receptor (EGFR) in ct-DNA originated from metastatic non-small-cell lung cancer (NSCLC) [138]. 

In addition, liquid biopsy techniques can overlap the existing diagnostic methodologies (combining liquid biopsy with the existing biomarkers and clinical analysis or imaging technologies) but can also substitute some, like invasive tissue biopsy. High-risk population groups can benefit from liquid biopsy as it can be used as a method for early cancer detection. This way, patients with predisposition for cancer can be tested for assessing their risk of disease development. In addition, patients can also be pharmacologically evaluated in order to assign the most efficient treatment regimens [139]. It also offers the benefits of fast detection and low-level biopsy-associated patient trauma [140]. As an example, taking solid tissue biopsy samples for brain and lung cancers is often painful and life-threatening [141], while in the case of pancreatic cancer, there is usually not enough tissue to confirm the diagnosis [142]. Another advantage of liquid biopsy of molecules is that they are dynamic, which permits real-time monitoring and can detect the heterogenicity of cancer, while tissue biopsy is static and only gives information about a specific tissue part in a defined moment [142]. Due to the tissue specificity of circulating molecules, it now presents as a growing market with great potential in medical diagnostics.

From an economic point of view, early and accurate cancer diagnosis can significantly reduce costs of cancer care. For instance, in 2017, the average cost for solid biopsy for lung cancer was $8869, while the total cost for a patient reached $37,745 because of 20% complications from needle biopsies [132,143]. Moreover, a transthoracic needle biopsy under computer tomography control may cost around $500, which is more than enough for a blood test as the current cost of single ct-DNA mutant detection with digital PCR is approximately $100 [99,133]. In addition, researchers from Johns Hopkins University have developed a multi-analysis blood assay that currently costs less than $500 [133,134].

### 4.2. Diagnostic Services for RNA Application

RNA-based technologies for prognostic, diagnostic and predictive therapies will be developing in the following years. A report published by Allied Market Research showed that by 2020, around 1.2 billion dollars had been spent on RNA diagnostics, and 6.8 billion dollars will be invested by 2028. The main targets would be diseases such as cancer, AIDS and different genetic disorders [144].

An indicator of the attractiveness of this field is acquisition of RNA-based companies by pharmaceutic giants, for example, of Santaris Pharma by Roche (2014) and of SiRNA Therapeutics by Merck (2007), followed by the acquisition of this division by Alnylam Pharmaceuticals (2014) [145].

The FDA-approved DNA-based liquid biopsy tests highly outnumber the RNA-based liquid biopsy assays. The assay that entered clinical practice detects the BCR–ABL1 fusion gene and was developed by several manufacturers (Table 5). The test is oriented towards monitoring patients with chronic myeloid leukaemia during treatment with tyrosine kinase inhibitors. Another test that entered clinical practice detects PCA3 in urine of men. The test helps doctors to decide whether to repeat prostatic biopsy or not.

### 4.3. Market Value of miRNA Diagnosis and Research

Cancer is the primary driver of the miRNA research field. This is supported by the data that today miRNA studies in cancer present 50% of the total field [147]. miRNAs are known for its involvement in carcinogenesis and their expressions in biological fluids have been shown and discussed earlier. Therefore, miRNAs have a great potential of being a nucleic acid marker for cancer detection and progression, as well as a possible target for new treatment approaches. Possible miRNA biomarkers have already been identified including miRNA-21, miRNA-126 and miRNA-155 for many solid cancers such as colon, prostate, lung and breast cancer [148,149,150,151,152,153,154]. Shortly, the adaption of miRNAs as biomarkers is the driving force for the diagnostic miRNA market value. For instance, in 2005 the market value of miRNA application in molecular diagnostics was worth $6.5 billion [155]. Fortune Business Insights reported market value of $215.4 million in 2019 [156]. Although the global market size of miRNA was expected to reach $626.27 million by 2025 [157], it was already valued at $854.6 million in 2020 [158]. In US North America, clinical trials using miRNA technology stood at $97.4 million in in 2019 [156]. Yet, the market value of RNA-based bio-pharmaceuticals was estimated to reach $1.2 billion by the end of 2020 [159] and 3.6 billion by 2028 [158] In 2020, the miRNA market was dominated by consumables (59.9%) that include detection kits, qPCR primers, and transfection reagents, to name a few. When it comes to the technologies, with share of over 32% PCR was leading the instruments segment [158]. From the consumers, academic and government research institutes accounted for the majority of the revenue share i.e., 54.2% in 2020.

This steady growth, estimated to be 18 to 20% within the next five years, is an indirect result of the increasing number of prevalence of chronic diseases including cancer. Moreover, the competition among companies to develop such tests will likely speed up the demand for their services in clinical settings. The biggest market is USA (45.8%), while Europe is the second largest holding 29.2% of global miRNA market share. Still, the miRNA market is growing fastest in Asia Pacific with a compound annual growth rate of 6% [160]. However, as the field is still developing it is difficult to precisely define its potential exact markets.

Smaller already established companies working and active promoting miRNA-based diagnostics for various disease types including test detecting single specific miRNAs are Interpace Diagnostics (ThyraMIR^®^/ThyGENX^®^) for Thyroid and pancreatic cancer risk classifier, Rosetta Genomics/Precision Therapeutics (miRview mets) and Genoptix (Reveal) are companies for detecting the origin of cancer and TAmiRNA (OsteomiR and ThrombomiR) detecting markers for osteoporosis or cardiovascular diseases, respectively. Furthermore, several other companies reaching pre-clinical and phase one testing. DiamiR is reaching the market using miRNA panel analysis for Alzheimer and brain disease diagnostics [145]. Quanterix/DestiNA Genomics using also developed an assay specifically using miRNA-423-5p to detect liver toxicity. An exosome-based liquid biopsy cancer diagnostic ExoDx Prostate test developed by Exosome Diagnostics of Cambridge, Massachusetts is available in the USA since 2016. Investments in the area reached $92 million soon after that [161]. And at last Hummingbird Diagnostics is currently developing and in phase 1 testing of detecting liquid biopsy miRNA signatures for the early detection of various diseases including non-small-cell lung carcinoma, melanoma, breast cancer, multiple sclerosis, Alzheimer, Parkinson, acute myocardial infarction, heart failure and inflammatory bowel disease [145]. Even though their tests are not commercially available yet, the company has 17 granted patents for blood expression profiling. Clinical validation of miRNA panels for the early diagnostics of different diseases by three European FP7-funded consortia, namely BestAgeing, RiskyCAD and EURenOmics, is currently ongoing.

### 4.4. Concerns Regarding the Clinical Aspect of Liquid Biopsy

Despite the several advantages of liquid biopsy, a few key issues, including standardization of pre-analytical sample handling (like blood collection, processing and storage), as well as robust workflows for probe extraction, quantification (digital PCR, NGS) and validation for molecular analysis of liquid biopsy samples must be guaranteed prior to market release [162,163,164]. Facilities will also have to improve data management and analysis, as well as the logistic needs (such as machinery) for tracking and running biological samples. Important aspect that needs to be discussed are the ethical issues. These must be solved prior market release. But also, the intellectual property has to be protected before commercialization of any of the liquid biopsy-based detection methods. In addition, the exact origin (is it correlated to cancer stage, is it from live/apoptotic cells, is it a communicating molecule looking for a potential metastatic place) of the analysed nucleic acids has to be clearly defined [165].

For clinical approaches, the most important criteria for RNA, particularly miRNA, as diagnostic and prognostic biomarker is high sensitivity and specificity to avoid false positive or negative diagnostic results. Furthermore, the appropriate biomarker for a specific cancer type needs to be both significantly differentially expressed and in correlation with the outcome of patients. It has to be considered that miRNAs in some cancer types are up regulated but the same miRNA can be downregulated in patients suffering from another cancer type [162,166]. Besides, the expression patterns are shown to be different during the cancer progression in, for example, NSCLC. Meaning that the contribution of miRNAs in various types and stages of cancers differs.

The last point to be considered for the transfer from basic research into routine laboratory diagnostics are costs. At the moment, liquid biopsy is too expensive when summarizing the costs for equipment, reagents, and the salary for professionals such as technical personnel, biochemists, bioinformatics specialists, nurses and physicians. Taken this factor into account, the current costs for liquid biopsies are much higher than those for comparable conventional biopsies.

## 5. Conclusions and Future Perspectives

In the latest decade, the potential role of RNA as cancer biomarker has revolutionized the development of clinic diagnostics. It has especially shown advantages in non-invasive methodology, high precision and sensitivity, detecting disease at early stage, therapy follow-up, real-time monitoring and learning of the heterogenicity of the tumour profile. The detection of circulating free RNA, particularly miRNAs in liquid biopsies can be used as prognostic, diagnostic and predictive biomarkers. Moreover, it could help to assist clinicians to make decisions on patients with cancer types difficult to diagnose. In spite of these important advantages, the main challenges nowadays in cf-RNA field include the low concentration and stability of some cf-RNAs. However, miRNAs are intrinsically more stable for clinical applications. Isolating miRNAs and also other RNAs from exosomes may be more valuable resource. However, currently there is no universally accepted gold standard method for cf-miRNA or exosome isolation. A difficult impediment in both analysis is namely co-isolation of contaminants, especially lipoproteins, which contain RNAs themselves and can interfere with further analysis.

Liquid biopsy will unlikely substitute current established diagnosing methods completely but can be very useful for early diagnosis or in cases where tissue is inaccessible or unavailable. Although they have the potential to be applied for personalized medicine, the commercialization process of liquid biopsy methods (based on the detection of ctRNA or exosomes) requires numerous time-consuming laboratory studies and rigorous clinical trials. The current gold standard method for miRNA quantification is qPCR. Novel methods as biosensors (known from blood glucose measurements or pregnancy tests) suppose a rapid, low-cost and easy-to-handle point of care testing for cf-RNA diagnostics. Once the protocols for circulating biomarkers are optimized, all validation and safety tests are passed, and approvals are obtained, they can have a significant impact on reducing the diagnostic costs. At the moment, the implementation of cf-RNA diagnostics in the clinic is rapidly increasing worldwide and billions of dollars are being invested in the field, and the amount is expected to rise in the following years.

## Figures and Tables

**Table 1:** Sources of liquid biopsy biomarkers and their terminology.

Terminology	Full Name and Brief Description
cf-DNA: distinguishing between both terms (cell-free DNA and circulating free DNA) is often not possible in literature.	Most of the time it means circulating free DNA. Degraded DNA fragments (50–200 base pairs) are released to the bloodstream.
	Cell-free DNA refers to the DNA which is not contained inside the cell nuclei. It is obtained by blood separation without cell lysis.
ct-DNA	Circulating tumour DNA: originates from tumour cells and is released to the bloodstream.
CTC	Circulating tumour cells: tumour cells released to the bloodstream.
cf-RNA: distinguishing between both terms (cell-free RNA and circulating free RNA) is even more complicated than with cf-DNA. The full names are often mixed and used indistinctly in literature.	Most of the times it means circulating free RNA. Degraded RNA fragments released to the bloodstream.
	Cell-free RNA: refers to the RNA not contained in the cell. Released to the bloodstream.
ccfNA	Circulating cell-free nucleic acids are a mixture of single- or double-stranded nucleic acids released into the blood plasma/serum by different tissues via apoptosis, necrosis and secretions.
EV	Extracellular vesicle: membrane particles derived from various cell types. Usually classified into exosomes, microvesicles, oncosomes and apoptotic bodies.
Exosome	The smallest extracellular vesicles (approximately 30–150 nm) released from cells. They are formed as intraluminal vesicles inside multivesicular bodies (MVB).
Small extracellular vesicle	According to MISEV guidelines 2018, other terms are suggested instead of exosomes. The terms can be based on different characteristics such as size, e.g., small and medium/large EVs together with the size range (e.g., <100 nM or <200 nM for small EVs)

**Table 2:** Methods for cf-RNA isolation.

Methods	Price	Duration	Throughput	Advantages	Disadvantages
Silica membranes	Low	1–2 h	High	Easy to use Efficient purification compared to the conventional methods Efficient and selective binding	Low volume Clogging the membrane Incomplete cellular lysis can lead to low yields Presence of contaminants
Magnetic beads and precipitation	Low	1–2 h	High	Easy to use Efficient purification compared to the conventional methods Efficient and selective binding No centrifugation need Virtually equipment-free process	Low volume Difficulty in automation
TRIzol reagent	Low	1–2 h	High	Easy to use Total RNA	Presence of contaminants Many small RNAs are lost

**Table 3:** Methods for exosomes isolation.

Method	Price	Duration	Throughput	Advantage	Disadvantage
Standard ultracentrifugation ^1^	High	3–4 h	Low	Easy to use Large volumes can be used	Presence of contaminants Time-consuming
Sucrose cushion ultracentrifugation ^1^	High	3–4 h	Low	Easy to use Large volumes can be used	Presence of contaminants Time-consuming
Sucrose gradient ultracentrifugation ^1^	High	≥16 h	Low	Easy to use Large volumes can be used	Presence of contaminants Time-consuming
Size-exclusion chromatography	Low	1 h	Intermediate	Easy to use	Presence of contaminants
Immunoaffinity	Medium	3 h	Intermediate	Easy to use High yield and specificity	No universal marker for exosomes
Precipitation	Low	1.5 ≥ 14 h ^2^	High	Easy to use	Presence of contaminants

^1^ The price highly depends on the usage frequency. ^2^ The time depends on the commercial kit.

**Table 4:** Methods for RNA quantification.

Method	Price	Duration	Performance	Advantages	Disadvantages
NGS-RNA Seq	Hundreds of dollars [99]	3–5 days [99]	Precise Maximum of 0.1% detection [100] To increase the sensitivity (reduce the background noise), you can incorporate UIMS (unique molecular identifiers)	Massive parallel Genome-wide RNA [100] High multiplexing capacity Possibility of identification of new variants CleanPlex^®^ is a highly scalable and ultrasensitive NGS amplicon sequencing technology [101]	Medium sensitivity Less sensitive than ddPCR Complex processing bioinformatics and computational resources [102] The error is dependent on the context and on technical parameters such as the length of the reading
miRNA sequencing	Hundreds of dollars [103]	1–2 weeks [103]	Precise	Sequence independence coverage in Philadelphia is now offering a microarray-based test (miRview Mets) to identify the tissue of origin in CUP Cases (unknown primary cancer) miRNA-SEQ method facilitates the sequencing of large pools of small RNAs in a single sequencing run [94]	Sequencing preparations involve creating libraries by cloning of DNA reverse-transcribed from endogenous small RNAs selected by column and gel electrophoresis Need of use of prediction software for identifying miRNAs mRNA targets [103]
Microarray	Hundreds of dollars [103]	2–4 days [103]	Precise Genome-wide RNA [104,105]	Open array—Thermo Fisher Scientific The best option if performing analysis of hundreds of probes	Short sequence specialized equipment [106]
Sanger Sequencing	High costs for a single experiment [100]	2–3 days preparation	Lower sensitivity, approximately a 15–10% [107]	Targeting smaller genomic regions in a larger number of samples Easy to use	Relatively low accuracy, precision and specificity [108] Purity and degradation rate of genetic material [109] Only sequences a single DNA fragment at a time Laborious cloning methods
RT-qPCR	Less than a hundred dollars	4–7 h	Sensitive [110] Detection limit is 1%	Well-stablished method Use of Taqman or FRET probes to gain specificity [104,111] Allows for multiplexing Use of a stem loop or poly A Use of Taqman or Molecular Beacon [112,113,114]	Necessity of the standard curve Limited multiplexing and genome-wide covering [102]
ddPCR	Less than a hundred dollars [99]	4–7 h [99]	Ultrasensitive [104,110] Technique used for specific RNA sequence, 0.01–0.001% limit detection [99]	Multiplexing Ultraprecision	Researchers can influence the distribution of the drops, and therefore the outcome Requires previous optimization Difficult multiplexing
BEAMing (beads, emulsion, amplification and magnet)	Hundreds of dollars [99]	2 days [99]	Ultrasensitive 0.01% limit detection [115,116]	Divide in so a way that each particle is attached to a magnetic bead like a microreactor Absolute quantification Detection of low frequencies	Requires previous optimization Difficult multiplexing Limited to predesigned variants [102]
CRISPR-Cas diagnostics (electrochemical biosensors for microRNA detection)	Low costs [117]	<4 h [117]	Precise Limit-of-detection: 2–10 pM [118]	Easily scalable [117] Easy-to-use, portable, multiplexed and amplification-free method for POCT [117,119] Cas13a-powered signal amplification [117,119]	Low sensitivity due to missing target amplification [119]

**Table 5:** FDA approved RNA-based tests for liquid-biopsy [146].

Trade Name	Manufacturer	Sample	Indication for Use	Method
Quantidex qPCR BCR-ABL IS Kit	ASURAGEN, Inc.	Whole blood	Monitoring patients with CML during treatment with tyrosine kinase inhibitors	RT-PCR detecting BCR-ABL1 and ABL1
Xpert BCR-ABL Ultra, GeneXpert Dx System, GeneXpert Infinity-48s and GeneXpert Infinity-80 Systems	Cepheid	Whole blood	Monitoring patients with CML during treatment with tyrosine kinase inhibitors	RT-PCR detecting BCR-ABL1 and ABL1
QXDx BCR-ABL %IS Kit for use on the QXDx Auto DG ddPCR System	Bio-Rad Laboratories, Inc.	Whole blood	Monitoring patients with CML during treatment with tyrosine kinase inhibitors	RT-PCR detecting BCR-ABL1 and ABL1
MRDx BCR-ABL Test, MRDx BCR-ABL Test Software	MolecularMD Corporation	Whole blood	Monitoring patients with CML during treatment with tyrosine kinase inhibitors	RT-PCR detecting BCR-ABL1 and ABL1
PROGENSA PCA3 assay	Gen-Probe, Inc.	Urine	To aid in the decision for repeated prostatic biopsy	Detecting PCA3 and PSA using Transcription Mediated Amplification and Hybridization Protection Assay (HPA) method
